# *Echinococcus Equinus* Found in Imported Donkeys (*Equus asinus*) From Central Asia

**DOI:** 10.1155/tbed/9570858

**Published:** 2026-05-30

**Authors:** Nannan Cui, Jun Xu, Jiyuan Wu, Suwen Wang, Ziman Lv, Ziqi Wang, Chenchen Jin, Peiyue Deng, Gang Liu, Jianping Cao, Yuanzhi Wang, Wenbo Tan

**Affiliations:** ^1^ Key Laboratory for Prevention and Control of Emerging Infectious Diseases and Public Health Security, The XPCC, School of Medicine, Shihezi University, Shihezi, Xinjiang Uygur Autonomous Region, China, shzu.edu.cn; ^2^ NHC Key Laboratory of Prevention and Treatment of Central Asia High Incidence Diseases, Shihezi University, Shihezi, Xinjiang Uygur Autonomous Region, China, shzu.edu.cn; ^3^ Urumqi Customs District P.R. China, Urumqi, Xinjiang, China; ^4^ NHC Key Laboratory of Parasite and Vector Biology, WHO Collaborating Centre for Tropical Diseases, National Institute of Parasitic Diseases, Chinese Center for Disease Control and Prevention (Chinese Center for Tropical Diseases Research), Shanghai, China

**Keywords:** cyst, *Echinococcus equinus*, imported donkeys, port

## Abstract

The zoonotic tapeworm *Echinococcus equinus* (G4) has been reported primarily in equines across Europe, Africa, and West Asia, but its presence in donkeys in Central Asia had not been documented. This knowledge gap is critical given the substantial and growing Kyrgyzstan–China trade of live donkeys. To assess the transboundary infection and associated risks, we conducted molecular surveillance on 1900 imported donkeys at a port slaughterhouse during November 2023–February 2024. A total of 52 hydatid cysts were collected from 18 infected animals. Protoscoleces were isolated from each cyst and examined microscopically for morphological observation and fertility assessment using Ponceau S staining. The protoscoleces were analyzed by PCR and sequencing of the *cox1* and *nad1* genes for species identification. The complete mitochondrial genome was sequenced on an Illumina NovaSeq 6000 platform, assembled de novo using SPAdes, and annotated with MITOS. Furthermore, we performed infection with 530–4440 protoscoleces via intraperitoneal injection in C57BL/6 mice. Our results identified *E. equinus* in liver and lung cysts from 0.9% (18/1900) donkeys by complete mitochondrial genome (13,722 bp; GenBank: PX243530). Strikingly, liver cysts developed in 3 out of 18 C57BL/6 mice after 180 day postinfection. This study provides the first evidence of *E. equinus* infection in imported donkeys from Central Asia.

## 1. Introduction

Echinococcosis, a zoonotic helminthiasis listed among neglected tropical diseases, remains a major public health concern across Western China, Central Asia, South America, the Mediterranean, and Eastern Africa [1]. The disease is caused by the metacestode (larval) stage of tapeworms belonging to the genus *Echinococcus* (Platyhelminthes: Cestoda: Cyclophyllidea: Taeniidae). This genus, within the *Echinococcus granulosus* sensu lato complex, encompasses several species and genotypes: *E. granulosus* sensu stricto (G1, G3), *E. equinus* (G4), *E. ortleppi* (G5), *E. canadensis* (G6/7, G8/10), and *E. felidis* [2–7]. Among these, *E. equinus* (G4) demonstrated a life cycle primarily involving equines (e.g., horses, donkeys, mules, and zebras) and canid carnivores (e.g., dogs, foxhounds, and jackals) [8–13]. Occasionally, it was also documented in a broader range of hosts, including lions, rhinoceros, and lemurs [13–15]. Geographically, *E. equinus* has been documented in equines across Europe, Africa, and Western Asia, including the United Kingdom, Ireland, Belgium, Switzerland, Italy, Spain, Namibia, Kenya, and Turkey, demonstrating strong host adaptation to equids [8–16]. Human and rodent infection occurred only accidentally [17–19].

Central Asia is a recognized hyperendemic focus for echinococcosis [20, 21]. Kyrgyzstan, whose livestock husbandry is economically critical, faces a severe public health burden from cystic and alveolar echinococcoses both for humans and livestocks [22, 23]. For example, the prevalence of *E. granulosus* in dogs was reported to be as high as 17.8% (83/466) in Southeastern Kyrgyzstan [22]. Occasionally, *E. equinus* was found in dogs in Kyrgyzstan [22]. Driven by demand for Ejiao (a herb, its main material comes from donkey skin), donkey importation from Kyrgyzstan to China was strengthened. Five hundred Kyrgyzstan donkeys in 2023 and 2904 donkeys in 2024 were imported into China [24]. However, the *Echinococcus* infection status of imported donkeys remains unknown. This study aimed to investigate the occurrence of *E. equinus* in donkeys imported from Kyrgyzstan by sequencing the complete mitochondrial genome and assess its ability to establish infection in a nonequid laboratory host (C57BL/6 mice).

## 2. Materials and Methods

### 2.1. Sample Collection

During November 2023–February 2024, abattoir surveillance was conducted on 1900 donkeys imported from Kyrgyzstan at a port slaughterhouse, Northwestern China (Supporting Information 1: Figure S1). A total of 52 hydatid cysts were collected from 18 infected animals. Of these, 45 cysts from 17 donkeys were located exclusively in the liver, while 7 cysts in one donkey were present in both the liver and lung. The cyst diameter ranged from 0.5–8 cm (Supporting Information 2: Figure S2A and B). Postmortem inspections were conducted onsite, following routine port quarantine protocols. The procedure included a systematic visual examination of the liver and lung surfaces for hydatid cysts. All collected cysts were transported to our laboratory (located in Shihezi City, about 1700 km distance) by car under 4°C conditions.

### 2.2. Isolation and Microscopic Examination of Protoscoleces

The protoscoleces (PSCs) from each hydatid cyst were centrifuged at 500 × *g* for 5 min at 4°C. The supernatant was discarded, and the pellet was resuspended in 30 mL of saline and washed three times. The individual sediments were transferred into 1.5 mL tubes, and 1 µL was microscopically examined for counting [25]. PSC fertility was determined using Ponceau S staining (Supporting Information 2: Figure S2C).

### 2.3. Infection of C57BL/6 Mice

Under germ‐free conditions, 21 specific pathogen‐free (SPF) female C57BL/6 mice (42 days old) were used in this study. Eighteen mice were intraperitoneally inoculated with *Echinococcus* PSCs (530–4440 protoscoleces per inoculum, 19.4%–83.2% fertility), and three received saline water as the negative controls (shown in Supporting Information 3: Table S1). All mice were humanely sacrificed at 180 days postinfection to assess the presence of hydatid cysts. Our experimental procedures complied with the guidelines of the Institutional Animal Care and Ethics Committee of Shihezi University of Medical Sciences (Approval Number A2022‐071‐01).

### 2.4. DNA Extraction and Molecular Detection

Genomic DNA was extracted from PSCs obtained from each of 52 hydatid cysts collected from the 18 infected donkeys using the TIANamp Genomic DNA Kit (TIANGEN, Beijing, China). A sheep cyst containing *E. granulosus* G1 served as a positive control [26], and double‐distilled H_2_O as the negative control. PCR amplification was performed targeting a 1610 bp fragment of the *cox1* gene and a 1010 bp fragment of the *nad1* gene [27]. The *cox1* gene was amplified using primers *cox1*‐F (5′‐AGTTACTGCTAATAATTTTGTGTCAT‐3′) and *cox1*‐R (5′‐ATGATGTAAAAGGCAAATAAACC‐3′), while *nad1* was amplified using *nad1*‐F (5′‐TAATGTTGATTATAGAAAATTTTCGTTTTACACGC‐3′) and *nad1*‐R (5′‐CACAATTTATTATATCAAAGTAACCTGC‐3′). Each 25 µL PCR reaction contained 12.5 µL of 2 × Taq PCR Master Mix (Vazyme, Nanjing, China), 0.5 µM of each primer, and 1.5 µL of template DNA. The thermal cycling conditions consisted of an initial denaturation at 95°C for 3 min, followed by 35 cycles of 95°C for 30 s, 55°C for 30 s, and 72°C for 90s, with a final extension at 72°C for 10 min. All 52 cyst samples were successfully amplified, and all PCR products were sequenced in bi‐directions. The PCR products were sequenced and compared with GenBank data using BLASTn (http://www.ncbi.nlm.nih.gov/BLAST/). Maximum likelihood phylogenetic trees were constructed in MEGA 7.0 based on the *cox1* and *nad1* gene sequences, employing the TN93+G+I and HKY + G substitution models, respectively, with nodal support assessed through 1000 bootstrap replicates (values ≥70% shown).

### 2.5. Mitochondrial Genome Sequencing, Assembly, and Annotation

Genomic DNA was extracted from protoscoleces of a representative *E. equinus*‐positive cyst using the TIANamp Genomic DNA Kit (TIANGEN, Beijing, China). After DNA quality verification, a sequencing library with an insert size of 350 bp was constructed using the Nextera XT DNA Library Preparation Kit (Illumina, San Diego, CA). Paired‐end sequencing (2 × 150 bp) was performed on an Illumina NovaSeq 6000 platform. Raw reads were filtered using fastp (https://github.com/OpenGene/fastp) with the following criteria: removal of reads with > 5% N bases, removal of reads where low‐quality bases (quality value ≤ 5) exceeded 50% of the read length, and removal of reads with adapter contamination. The clean data were de novo assembled using SPAdes v.3.14.1 with multiple k‐mer settings (21, 45, 65, 85, and 105). The resulting assembly graph was visualized and validated using Bandage software to confirm the complete circular mitochondrial genome. The assembled genome was annotated using the MITOS2 webserver (http://mitos.bioinf.uni-leipzig.de/index.py), followed by manual correction of annotation results. Finally, a circular map of the mitochondrial genome was generated using Organellar Genome DRAW (OGDRAW) v1.2.

## 3. Results

### 3.1. Identification of Echinococcus Species

**Figure 1:**
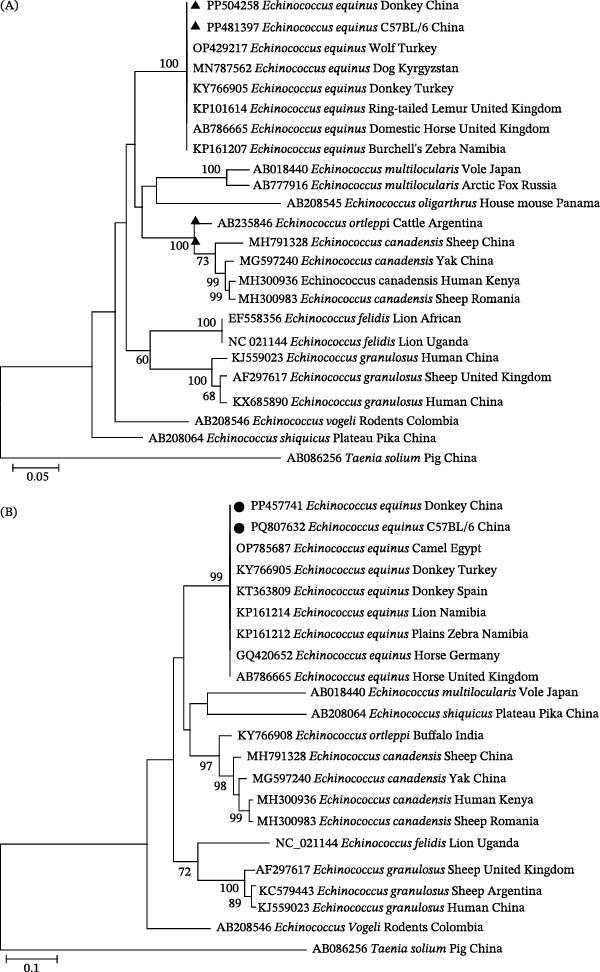
Phylogenetic analysis of *Echinococcus equinus* isolates based on mitochondrial gene sequences. (A) Maximum likelihood tree reconstructed from the *cox1* gene (1611 bp) using the Tamura–Nei model with gamma‐distributed rates and invariant sites (TN93 + G + I). (B) Maximum likelihood tree reconstructed from the *nad1* gene (1037 bp) using the Hasegawa–Kishino–Yano model with gamma‐distributed rates (HKY + G). Both trees were generated in MEGA 7.0 with 1000 bootstrap replicates; bootstrap values ≥70% are shown at nodes. Sequences obtained in this study are marked with a black triangle (A, *cox1*) and a black circle (B, *nad1*). GenBank accession numbers, host species, and geographic origins are indicated for all reference sequences.

Based on *cox1* and *nad1* sequences, BLASTn and phylogenetic analyses indicated that all 52 cyst samples were identified as *E. equinus* (G4 genotype). Both *cox1* and *nad1* sequences were 100% identical among infected individuals and showed 100% identity to *E. equinus* sequences previously reported from dogs in Kyrgyzstan (MN787562), wolves and donkeys in Turkey (OP429217, KY766905), plains zebras in Namibia (KP161207), and horses in England (AB786665) (Figure 1A), as well as donkeys in Turkey and Spain (KY766905, KT363809), plains zebras and lions in Namibia (KP161212, KP161214), camels in Egypt (OP785687), and horses in Germany (GQ420652) and England (AB786665) (Figure 1B). The nucleotide sequences generated in this study were deposited in GenBank with accession numbers PP504258 (*cox1*, donkey), PP457741 (*nad1*, donkey), PP481397 (*cox1*, mouse), and PQ807632 (*nad1*, mouse).

### 3.2. Mitochondrial Genome Characteristics of *E. equinus*

**Figure 2:**
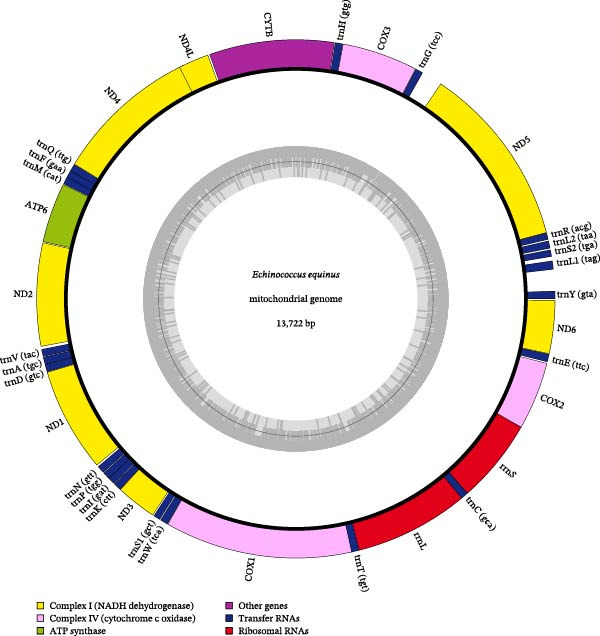
Annotation map of the *Echinococcus equinus* mitochondrial genome. Genes inside the circle are transcribed in the counterclockwise direction, while those outside are transcribed in the opposite direction. Genes are color‐coded by functional category. The gray histogram inside the ring displays the GC content, with the central gray line representing the 50% threshold.

The complete mitochondrial genome of the *E. equinus* isolates was successfully sequenced, assembled, and annotated from a single representative cyst obtained from one of the infected donkeys. The final assembly yielded a single, circular double‐stranded DNA molecule of 13,722 bp (Figure 2), which was 99.98% (13512/13515) identical with *E. equinus* from horses in the United Kingdom. The individual nucleotide frequencies were determined as follows: A: 20.01%, T: 47.90%, C: 7.85%, and G: 24.24%. Annotation of the genome identified the full complement of 37 mitochondrial genes, which is typical for taeniid cestodes (Supporting Information 4: Table S2).

### 3.3. Infection of C57BL/6 Mice With *E. equinus*

**Figure 3:**
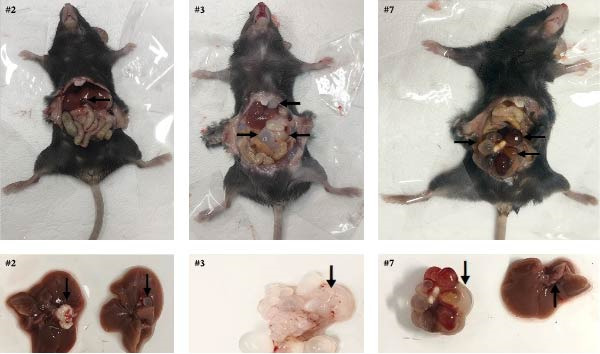
Pathological findings in C57BL/6 mice 180 days postinfection. Cysts were shown with arrows. Both mice #3 and #7 had cysts in their liver and intestine, while mouse #2 had cysts in its liver.

Hydatid cysts developed in 16.67% (3/18) of mice after 180 days postinfection (Figure 3). One mouse (#2) harbored a cyst (~1 cm diameter) with thick and opaque cyst walls in the liver, while two mice (#3 and #7) each exhibited cysts (≤ 0.5 cm diameter) in both the liver and intestinal mucosa.

## 4. Discussion

This study provides the first confirmation of *E. equinus* in donkeys imported from Kyrgyzstan by veterinary clinics and complete mitochondrial genome sequencing. China’s donkey inventory has collapsed from over 11 million in the 1990s to ~1.46 million in 2023 due to increasing Ejiao industry demand [28, 29]. This deficit forces reliance on imports from Africa and Central Asia [30, 31]. Our findings offer direct evidence that this high‐stakes and supply‐constrained trade not only represents an economic activity but also constitutes a tangible pathway for infectious agent spillover. In this study, 0.9% (18/1900) Kyrgyzstan‐imported donkeys were infected with *E. equinus*. This prevalence is likely an underestimation as effective serological diagnostic tools for detecting *Echinococcus* infections in equids are currently lacking, clinical signs are often absent, and ultrasound examination is unsuitable for routine customs quarantine, especially for large‐scale slaughtered animals.

Previous studies have established that a high‐dose intraperitoneal inoculation of highly viable *E. equinus* protoscoleces (3000–4000 per individual) leads to robust cyst formation in BALB/c mice [32]. In this study, 530–4440 protoscoleces with 19.4%–83.2% fertility were used to challenge 18 C57BL/6 mice (shown in Supporting Information 3: Table S1), only three mice were successfully infected, and liver and/or hepatic‐pulmonary cysts occurred. The lowest doses (1210 protoscoleces with 33.9% fertility) could infect C57BL/6 mice and cause liver and/or intestinal mucosa cysts. This finding provides direct experimental evidence for *E. equinus* infection in nonequine intermediate hosts besides red‐ruffed lemurs and human beings [15, 18, 19].

Previously, *E. equinus* infections were documented in a dog in Kyrgyzstan and in a patient in Uzbekistan [17, 19]. In this study, we first confirm *E. equinus* in Kyrgyzstan‐imported donkey. Globally, *E. equinus* infects a broad range of carnivores (e.g., dogs, lions, black‐backed jackals, and foxhound) and equids (e.g., mules, Burchell’s zebras, horses, and mules) [8–16]. Our finding enriches the cumulative evidence across dogs, human beings, and donkeys in Central Asia. Although *E. equinus* is considered less frequently zoonotic than *E. granulosus* sensu stricto, humans infected with *E. equinus* were occasionally reported [17–19]. In Kyrgyzstan, over 40% of the workforce is engaged in agriculture and livestock production [33]. The transmission cycle from infected dog to donkey and farmer shouldn’t be underscored.

The economic implications of our findings are substantial. China is the world’s largest producer of Ejiao [34]. With donkey inventories declining from over 11 million in the 1990s to ~1.46 million in 2023, China has become heavily dependent on imports to sustain production. Historically, Africa served as the primary source, such as Kenya, Ethiopia, and Niger [34]. Recently, donkey demand turns to South America and Asian. In this study, *E. equinus* was prevalent in 0.9% of Kyrgyzstan‐imported donkeys, although the data were underscored. This finding indicates it is critical to develop suitable diagnostic methods for *E. equinus*, especially in customs quarantine.

Several limitations should be acknowledged. First, abattoir surveillance relied on visual inspection and targeted organ slicing, which may underestimate true prevalence by missing small or deeply embedded cysts. Second, the validated serological assays are lacking for large‐scale equids for *E. equinus* especially in customs quarantine. Third, the mouse infection experiment was a preliminary assessment of cross‐species infectivity rather than a perfect infection model.

In conclusion, this study provides three key advances: (i) the first evidence of *E*. *equinus* infection was confirmed in donkeys imported from Kyrgyzstan; (ii) C57BL/6 mice infection partially succeeded under laboratory conditions; and (iii) genetic divergence is little between *E*. *equinus* strains from Europe and Central Asia by sequencing the full‐length mitochondrial genome. This work focuses on typical transboundary case in imported donkeys, which indicates that abattoir surveillance is vital, especially for ignored or isn’t‐easy‐found infectious diseases.

## Funding

This work was supported in part by Open Project of the NHC Key Laboratory of Parasitic Pathogen and Vector Biology (Grant NHCKFKT2024‐2), the Key Scientific & Technological Project of XPCC (Grant 2025AA018), the National Natural Science Foundation of China (Grant 82260410), the Science & Technology Innovation Team Project of TIANSHAN Elite (Grant 2023TSYCTD0020), and the Open Subject of Central Asia High Incidence Disease Control Key Laboratory of National Health Commission (Grant KF202404).

## Ethics Statement

All procedures were verified according to the guidelines of the Institutional Animal Care and Ethics Committee of Shihezi University of Medical Sciences (Approval Number A2022‐071‐01).

## Conflicts of Interest

The authors declare no conflicts of interest.

## Supporting Information

Additional supporting information can be found online in the Supporting Information section.

## Supporting information


**Supporting Information 1** Figure S1: Map of the China–Kyrgyzstan border study area.

**Supporting Information 2** Figure S2: Clinical appearance of cyst from donkey livers and lungs, and protoscoleces observation of *Echinococcus equinus*. S2A: a cyst with 8 cm diameter from donkey liver; S2B: cysts found in a hepatic‐pulmonary case; and S2C: photomicrograph of protoscoleces stained by Ponceau S.

**Supporting Information 3** Table S1: Survival status and cyst localization in mice after *Echinococcus* protoscoleces inoculation.

**Supporting Information 4** Table S2: Gene content of the Echinococcus equinus mitochondrial genome.

## Data Availability

The datasets generated for this study can be found in the GenBank (Donkey *cox1* gene: PP504258; C57BL/6 *cox1* gene: PP481397; Donkey *nad1* gene: PP457741; C57BL/6 *nad1* gene: PQ807632; complete mitochondrial genome: PX243530).
