# Supplementary material for: Echinococcus Equinus Found in Imported Donkeys (Equus asinus) From Central Asia
Source: Transbound Emerg Dis. 2026 May 30;2026:9570858. doi: 10.1155/tbed/9570858 (PMC13239184; doi:10.1155/tbed/9570858)
Supplement: Supplementary file 1 — Supporting Information 1 Figure S1: Map of the China–Kyrgyzstan border study area. [file TBED-2026-9570858-s002.pdf]

***Supporting Informations 1*: Figure. S1. Map of the China-Kyrgyzstan border study area**

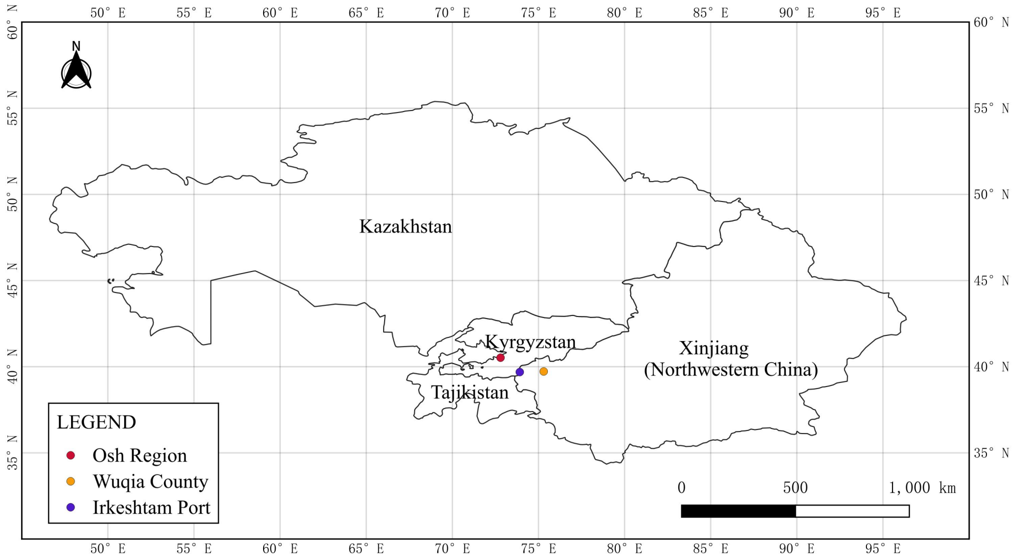

Location	Latitude (°N)	Longitude (°E)	Description
Osh Region	40.5167	72.8000	Source region of imported donkeys in Kyrgyzstan
Wuqia County	39.7179	75.3059	Location of port slaughterhouse in Xinjiang, China
Irkeshtam Port	39.6833	73.9167	Major border crossing point between the two countries
