# Supplementary material for: Echinococcus Equinus Found in Imported Donkeys (Equus asinus) From Central Asia
Source: Transbound Emerg Dis. 2026 May 30;2026:9570858. doi: 10.1155/tbed/9570858 (PMC13239184; doi:10.1155/tbed/9570858)

***Supporting Informations 2*: Figure. S2.**

Clinical appearance of cyst from donkey livers and lungs, and protoscolices observation of *Echinococcus equinus*. **S2. A**: a cyst with 8 cm diameter from donkey liver; **S2. B**: cysts found in a hepatic-pulmonary case; and **S2. C**: photomicrograph of protoscoleces stained by Ponceau S.

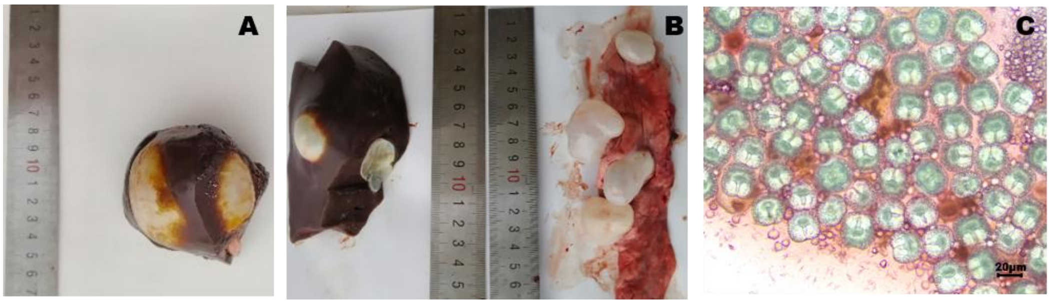

Supplement: Supplementary file 2 — Supporting Information 2 Figure S2: Clinical appearance of cyst from donkey livers and lungs, and protoscoleces observation of Echinococcus equinus. S2A: a cyst with 8 cm diameter from donkey liver; S2B: cysts found in a hepatic‐pulmonary case; and S2C: photomicrograph of protoscoleces stained by Ponceau S. [file TBED-2026-9570858-s003.pdf]
