# Supplementary material for: Echinococcus Equinus Found in Imported Donkeys (Equus asinus) From Central Asia
Source: Transbound Emerg Dis. 2026 May 30;2026:9570858. doi: 10.1155/tbed/9570858 (PMC13239184; doi:10.1155/tbed/9570858)
Supplement: Supplementary file 3 — Supporting Information 3 Table S1: Survival status and cyst localization in mice after Echinococcus protoscoleces inoculation. [file TBED-2026-9570858-s004.pdf]

Supporting Information 3**: Table S1.** Survival status and cyst localization in mice after *Echinococcus* protoscolices inoculation.

Mouse ID	Protoscolices	Fertility [a]/%	Result	
			Survival status	Location of cysts
#1	4200	75.0	Death (3 days post infection)	/
#2	3800	42.1	Survival	liver
#3	1440	35.4	Survival	liver and intestine
#4	4100	79.0	Death (6 days post infection)	/
#5	650	76.9	Survival	No cyst
#6	2730	19.4	Survival	No cyst
#7	1210	33.9	Survival	liver and intestine
#8	530	81.1	Survival	No cyst
#9	1240	45.2	Death (5 days post infection)	/
#10	900	80.6	Survival	No cyst
#11	900	80.6	Death (4 days post infection)	/
#12	1650	30.3	Survival	No cyst
#13	4120	72.8	Death (4 days post infection)	/
#14	4440	76.9	Survival	No cyst
#15	1410	31.2	Death (3 days post infection)	/
#16	1620	32.1	Death (7 days post infection)	/
#17	1010	83.2	Survival	No cyst
#18	2100	37.6	Death (5 days post infection)	/

[a] Protoscolex fertility was determined by Ponceau S staining and direct counting.
