# Supplementary material for: Echinococcus Equinus Found in Imported Donkeys (Equus asinus) From Central Asia
Source: Transbound Emerg Dis. 2026 May 30;2026:9570858. doi: 10.1155/tbed/9570858 (PMC13239184; doi:10.1155/tbed/9570858)
Supplement: Supplementary file 4 — Supporting Information 4 Table S2: Gene content of the Echinococcus equinus mitochondrial genome. [file TBED-2026-9570858-s001.pdf]

***Supporting Information 4*: Table S2.** Gene content of the *Echinococcus equinus* mitochondrial genome

Start	End	Length	Strand	Gene
1	66	66	+	trnY (gta)
250	322	73	+	trnL1 (tag)
359	416	58	+	trnS2 (tga)
430	493	64	+	trnL2 (taa)
506	563	58	+	trnR (acg)
565	2136	1572	+	ND5
2315	2379	65	+	trnG (tcc)
2383	3030	648	+	COX3
3033	3098	66	+	trnH (gtg)
3104	4171	1068	+	CYTB
4187	4447	261	+	ND4L
4408	5667	1260	+	ND4
5671	5732	62	+	trnQ (ttg)
5732	5794	63	+	trnF (gaa)
5791	5856	66	+	trnM (cat)
5862	6374	513	+	ATP6
6383	7264	882	+	ND2
7289	7351	63	+	trnV (tac)
7356	7419	64	+	trnA (tgc)
7420	7484	65	+	trnD (gtc)
7488	8381	894	+	ND1
8398	8463	66	+	trnN (gtt)
8470	8532	63	+	trnP (tgg)
8532	8595	64	+	trnI (gat)
8601	8664	64	+	trnK (ctt)
8667	9014	348	+	ND3
9026	9084	59	+	trnS1 (gct)
9093	9159	67	+	trnW (tca)
9163	10770	1608	+	COX1
10765	10829	65	+	trnT (tgt)
10830	11808	979	+	rrnL
11809	11870	62	+	trnC (gca)
11871	12591	721	+	rrnS
12592	13173	582	+	COX2
13186	13252	67	+	trnE (ttc)
13256	13711	456	+	ND6
